# Beneficial effects of premeal almond load on glucose profile on oral glucose tolerance and continuous glucose monitoring: randomized crossover trials in Asian Indians with prediabetes

**DOI:** 10.1038/s41430-023-01263-1

**Published:** 2023-02-02

**Authors:** Seema Gulati, Anoop Misra, Rajneesh Tiwari, Meenu Sharma, Ravindra M. Pandey, Ashish Datt Upadhyay, Hem Chandra Sati

**Affiliations:** 1 Diabetes Foundation (India), New Delhi, India; 2 National Diabetes, Obesity and Cholesterol Foundation (N-DOC), New Delhi, India; 3 Center of Nutrition & Metabolic Research (C-NET), New Delhi, India; 4 Fortis C-DOC Centre for Excellence for Diabetes, Metabolic Disease, and Endocrinology, New Delhi, India; 5 All India Institute of Medical Sciences, New Delhi, India

**Keywords:** Lifestyle modification, Diabetes

## Abstract

**Background:**

Rapid conversion from prediabetes to diabetes and frequent postprandial hyperglycemia (PPHG) is seen in Asian Indians. These should be the target of dietary strategies.

**Objectives:**

We hypothesized that dietary intervention of preloading major meals with almonds in participants with prediabetes will decrease overall glycemia and PPHG.

**Design:**

The study included two phases: (1) an oral glucose tolerance test (OGTT)-based crossover randomized control study, the effect of a single premeal almond load (20 g) given before OGTT was evaluated (*n* = 60, 30 each period). (2) The continuous glucose monitoring system (CGMS)-based study for 3 days including premeal almond load before three major meals was a free-living, open-labeled, crossover randomized control trial, where control and premeal almond load diets were compared for glycaemic control (*n* = 60, 30 in each period). The study was registered at clinicaltrials.gov (registration no. NCT04769726).

**Results:**

In the OGTT-based study phase, the overall AUC for blood glucose, serum insulin, C-peptide, and plasma glucagon post-75 g oral glucose load was significantly lower for treatment vs. control diet (*p* < 0.001). Specifically, with the former diet, PPHG was significantly lower (18.05% in AUC on OGTT, 24.8% at 1-h, 28.9% at 2-h post OGTT, and 10.07% during CGMS). The CGMS data showed that premeal almond load significantly improved 24-glucose variability; SD of mean glucose concentration and mean of daily differences. Daily glycaemic control improved significantly as per the following: mean 24-h blood glucose concentration (M), time spent above 7.8 mmol/L of blood glucose, together with the corresponding AUC values. Premeal almond load significantly decreased following: overall hyperglycemia (glucose AUC), PPHG, peak 24-h glycaemia, and minimum glucose level during night.

**Conclusion:**

Incorporation of 20 g of almonds, 30 min before each major meal led to a significant decrease in PPHG (as revealed in OGTT-based study phase) and also improved insulin, C-peptide, glucagon levels, and improved glucose variability and glycemic parameters on CGMS in participants with prediabetes.

**Clinical trial registry:**

The study was registered at clinicaltrials.gov (registration no. NCT04769726).

## Introduction

Type 2 diabetes (T2D) is rapidly increasing in India due to multiple factors; predominant among them are dietary and lifestyle transitions [1, 2]. There is a faster rate of conversion from prediabetes to diabetes in Asian Indians, and the reversal of prediabetes to normal glucose regulation is more difficult than in whites [3, 4]. Pattern of hyperglycemia in Asian Indians with T2D is characterized by the predominance of postprandial hyperglycemia (PPHG). In a study comparing Asian Indians with populations from four other regions globally, the former population showed the highest postprandial blood glucose (PPBG) levels [5]. Furthermore, Dickinson et al. [6] showed that lean, healthy South Asians exhibited higher postprandial glycemic excursions and lowest insulin sensitivity for similar dietary carbohydrate load as compared to European Caucasians, Chinese, and Arabic Caucasians.

Exaggerated postprandial spikes in blood glucose generate excess free radicals that can trigger inflammation, endothelial dysfunction, and sympathetic hyperactivity [7]. Such recurring postprandial metabolic dysregulation increases the risk for atherosclerosis. PPHG has been shown to increase the risk for cardiovascular disease (CVD) and diabetes-related complications [8]. Furthermore, post-lunch blood glucose levels predicted cardiovascular events in patients with T2D after a 5-year follow-up (*n* = 529) [9]. Finally, even in persons with normal glucose tolerance, cardiovascular risk has been shown to increase according to PPBG [10].

Our group has an interest in research on tree nuts and has conducted two previous trials. In the first trial, we showed that pistachio nuts, incorporated in the diet as 20% energy, resulted in improvement of multiple cardiometabolic factors including fasting blood glucose (FBG) in subjects with metabolic syndrome [11]. In the second study, the intervention of almonds as a snack (not before meals) in patients with T2D led to an improvement in glycosylated hemoglobin (HbA1C) [12]. In these studies, the effect on postprandial blood glucose levels was either not significant [11] or was not evaluated [12].

Based on the above discussion, we planned to research the effect of the ingestion of almonds before major meals (breakfast, lunch, and dinner) on PPBG in Asian Indians with prediabetes. To date, only one study is available in which postprandial blood glucose excursions after (30 min before the major meals) premeal almond load were evaluated. In this study, Crouch et al. [13] included 20 participants (of various ethnicities in the USA with prediabetes) who showed that a preload of 15 g of almonds decreased 1-h postprandial hyperglycemia by 19.4%. Of note, the study by Crouch et al. [13] wa*s* conducted on 20 participants of mixed ethnicity and focused only on blood glucose levels on the oral glucose tolerance test (OGTT).

We hypothesized that dietary intervention with almonds 20 g before OGTT and before meals (60 g per day, 20 g before breakfast, lunch, and dinner assessed through CGMS) will decrease the glucose and insulin excursions after meals, and thus reduce overall hyperglycemia and improve glucose-insulin metabolism.

## Methods

This study was conducted according to the guidelines laid down in the Declaration of Helsinki and Ethical Guidelines for Biomedical Research for Human Participants as enunciated by the Indian Council of Medical Research, New Delhi, India. All procedures involving human participants were approved by an independent review committee, “Ethics Committee for Human Research.” Written informed consent forms, approved by the ethics committee, were signed by the participants. The study was initiated in January 2018 and was completed in June 2021. The study was registered at clinicaltrials.gov (registration no. NCT04769726, available at https://register.clinicaltrials.gov/prs/app/action/SelectProtocol?sid=S000AO3N&selectaction=Edit&uid=U0000VVL&ts=4&cx=-hrv1fh).

A total of 60 participants (27 males and 33 females), aged between 18 and 60 years and confirmed with prediabetes were recruited for the study. In total, we screened 1317 participants, out of which 1171 were screen failures and did not fulfill the inclusion/exclusion criteria. Sixty participants gave their consent for the study and were recruited. Prediabetes was diagnosed on OGTT according to the following criteria: fasting blood glucose 5.6 and 7 mmol/L and 2-h plasma glucose (after ingestion of 75-g anhydrous oral glucose mixed in 200 mL water) 7.8 and 11.1 mmol/L or 2-h plasma glucose 7.8 and 11.1 mmol/L [14]. Those with known diabetes, acute infections, advanced end-organ damage, history of pancreatitis, significant renal and liver disease, recent (<3 months) changes (≥5%) in weight and/or on weight-changing medications, any known allergy to nuts, or have uncontrolled hypertension or hypothyroidism were excluded from the study. Participants were instructed not to consume any other nuts in the Intervention arm and nuts in any form in the control diet group. This crossover study was conducted at National Diabetes, Obesity and Cholesterol Foundation (http//n-doc.org.in), Delhi, India. The nature of the study objective was supported by a crossover study design. As this study design gave an advantage in terms of the number of participants and also the short-term nature of the study ensured that the participants’ condition resumed back after the washout period without any carryover effect.

**Fig. 1:**
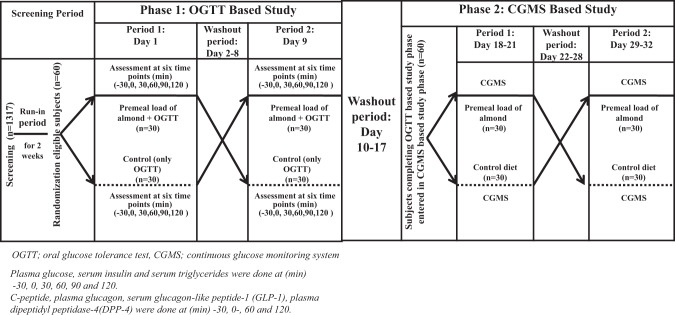
Study flowchart shows a 32 days plan for oral glucose tolerance (OGTT)-based study and continuous glucose monitoring system (CGMS) study phases. After initial screening, eligible participants were randomized to a premeal almond load treatment-control sequence or to the control-treatment sequence after a run-in period of 2 weeks. Intravenous blood was withdrawn at −30, 0, 30, 60, 90, and 120 min in each period. After completion of the OGTT-based study phase, participants entered the CGMS-based study phase. The CGMS device was inserted in the subcutaneous tissue of the lateral abdominal wall for 3 days. Participants were asked to record activity, mealtime, and premeal almond load timings, which was indicated in Supplementary Fig. 1. After 3 days CGMS was removed, and participants were given a normal diet from 22 to 28 days followed by crossover and application of CGMS for another 3 days. There was no participant dropout in the study.

In the OGTT-based study phase (evaluation on OGTT) after a run-in period of 2 weeks, participants were randomized to the control-treatment sequence or treatment-control sequence and then crossed over after a washout period of 7 days (Fig. 1). The effect of a single premeal almond load before OGTT (*n* = 60, 30 in each period) was evaluated. Participants on the treatment diet were given a premeal almond load of 20 g of almonds 30 min prior to ingestion of 75 g of glucose, while those on the control diet were not given any food/almonds before OGTT.

Intravenous blood samples were collected at (min): −30, 0, 30, 60, 90, and 120. A crossover of these participants was done and OGTT was repeated in a similar manner. (Fig. 1)

Plasma glucose level, serum insulin levels, and serum triglyceride levels were estimated at (min) −30, 0, 30, 60, 90, and 120. C-peptide, plasma glucagon, serum glucagon-like peptide-1 (GLP-1), and plasma dipeptidyl peptidase-4 (DPP-4), levels were assessed at (min) −30, 0, 60, and 120. Assays were done in the following manner: blood glucose and serum triglycerides (enzymatic colorimetric test, Cobas Integra analyzer, Roche Diagnostics GmbH, Mannheim, Germany), serum insulin levels (Chemiluminescence immunoassay, Roche Diagnostics GmbH, Mannheim, Germany), GLP-1 (ELISA assay, Epitope Diagnostics, Inc. San Diego, CA, USA), plasma DPP-4 levels (ELISA assay, Boster Biological Technology, Pleasanton, USA), plasma glucagon (ELISA assay, Sincere Biotech Co. Ltd. Beijing, China), and C-peptide (Electrochemiluminescence immunoassay, Roche Diagnostics GmbH, Mannheim, Germany). In the OGTT-based study phase, participants on the treatment diet were given 20 g of almonds 30 min before drinking 75 g of glucose dissolved in 200 mL of water. While participants on the control diet were not given any food item before OGTT. The nutritional information on almonds is provided in Supplementary Table 1.

The continuous glucose monitoring system (CGMS)-based study phase, using CGMS, was a free-living, open-labeled, crossover randomized control trial, where control and premeal almond load diets were compared in 60 participants with prediabetes (*n* = 30 each period). In this phase, the effect of premeal almond load on blood glucose profile was monitored for three days using CGMS (see details below). Participants on the treatment diet received a premeal almond load for 3 days and then studied without premeal almond load after crossover. A washout period of 7 days separated the crossover period (Fig. 1).

The iPro2 CGM device (iPro2 Professional CGM; Medtronic MiniMed, Northridge, CA) is based on the principle of electrochemical detection of blood glucose in subcutaneous interstitial fluid. The CGM sensor was inserted in the subcutaneous tissue of the lateral abdominal wall. The CGMS records and stores 288 glucose readings during a 24-h period as the participants perform the routine daily activities. The sensor was calibrated using finger-prick capillary blood glucose measurements taken at fasting, pre- and post-meals (breakfast, lunch, and dinner) periods by using a glucose meter (Contour Plus One blood glucose meter, Ascensia Diabetes Care Holdings AG, Switzerland). The iPro2 device was worn for 3 complete days. The following day participants returned the iPro2 recorder for upload of the data to a web-based software (CareLink iPro software, MMT-7340, Medtronic MiniMed, Northridge, CA, USA) which provided a summary of the glucose levels over 3 days.

Definitions of parameters derived from CGMS are as follows:

1. The parameters used for glycaemic variability: mean amplitude of glucose excursions (MAGE) (mmol/L), the standard deviation of mean 24-h CGMS blood glucose readings (mmol/L), and mean of daily differences (MoDD).
2. Parameters used for glycaemic control/extent of glycemia: mean 24-h blood glucose reading (M) (mmol/L), time (min) spent in the ideal range (3.9–7.8 mmol/L), AUC ideal range (3.9–7.8 mmol/L) [14], time spent above 7.8 mmol/L per day (min), AUC > 7.8 mmol/L per day, time above 11.1 mmol/L per day (min), Peak 24-h glycaemia reading (mmol/L), minimum blood glucose reading during the night (mmol/L), PPHG AUC pp (mmol/L), overall hyperglycemia AUC total (mmol/L) and basal hyperglycemia AUC (mmol/L) (AUC total-AUC pp) above 7.8 mmol/L.

The above parameters were calculated using the software as mentioned using different formulae (Supplementary file 1).

Participants on the control diet consumed a standard diet advised according to guidelines for Asian Indians in India [15]. Instructions were given verbally and in written form and were discussed in detail individually and during group meetings. The general qualitative recommendations for both control and treatment diets were to consume a diet rich in vegetables and fruits; select whole-grain, high-fiber foods; select fat-free or low-fat dairy products; limit foods containing partially hydrogenated vegetable oils; curtail consumption of sugar-sweetened beverages and foods with added sugar; cut down on salt; and limit alcohol intake. Participants on the treatment diet were given pre-weighed packets of 20 g of almonds and it was emphasized that participants consume a packet of 20 g of almonds 30 min before each major meal (breakfast, lunch, and dinner) for 3 days in a CGMS-based study. Those on the control diet were not given any food items before the major meals. The verbal compliance check showed that they all followed. Dietary data were collected using the 24-h dietary recall method (analyzed using software, “DietCal” version 5.0; Profound Tech Solution; http://dietcal.in/) based on values from the Nutritive Value of Indian Foods. Furthermore, a food frequency questionnaire (FFQ) was used to check the pattern of food consumption before and after the run-in period.

Dietary composition (% energy) was as follows—control diet: carbohydrates, 50; fat, 35; and protein,15; and treatment diet (with premeal almond load): carbohydrates, 49; fat, 32; and protein, 19. The macronutrient intake of the treatment and control diet has been provided in Supplementary Tables 2 and 3. All data were recorded in a call log on days 18–21 and days 29–32 in CGMS-based study. The diet and exercise records were maintained in a compliance log, which was prepared and given to all participants. In this log, participants recorded their food intake, physical activity, and blood glucose levels. Based on this log, compliance to and acceptability of the diet was good (90%), as almonds were convenient to carry to the workplace, tasted good, and were generally considered nutritious food. Diet and exercise status were assessed telephonically daily for three days and through text messages. There was no difference in compliance to diet and exercise between the participants on premeal almond load treatment and control diets. We observed that 90% of the meals (range 85–95% of meals) were as per the dietary advice given to study participants on both diets. The compliance checks were done on an everyday basis. All the participants were given a glucose meter to monitor their blood glucose levels during the CGMS-based study phase. Betterment of blood glucose readings post meals was a motivational factor leading to good compliance in many participants. No adverse effect was reported.

Calculation of sample size: based on previous studies [13], the anticipated sample size has been calculated as a mean level post-test meal of blood glucose (per unit of time during 150 min duration) on OGTT as 6.1 mmol/L and on control diet as 7.2 mmol/L with pooled standard deviation as 2.2 mmol/L, with 95% confidence level and 90% power in a two-period crossover mode, we required a minimum of 22 participants who would receive the intervention followed by control or control followed by intervention as per the randomization. However, we have included 30 participants in each of the two sequences so as to have 60 data points on the intervention diet and 60 data points on the control diet at the end of the second period. For phase 2 of the study, in the absence of any preliminary data, we were unable to do any formal sample size determination.

Randomization: Sequence (premeal almond load treatment followed by standard OGTT or standard OGTT followed by premeal almond load treatment meal) of random numbers were generated by the statistician, not involved in the trial STATA 15.0 statistical software (College Station, Texas, USA). The study Investigator enrolled the participants and assigned participants to the sequence of interventions. This process was followed for both study phases. The trial was stopped after the last follow-up of the last participant enrolled in phase 2 of the study. For every participant, the allocated random number was placed in an opaque envelope. Only the sequence number was mentioned on the cover of the envelope. The envelope was opened at the enrollment for every participant. In these short-term studies, there was no dropout or loss to follow-up. Sixty participants were enrolled in the study and 60 only completed the study.

### Statistical analysis

Data were recorded in a predesigned proforma and managed on an Excel spreadsheet. All the entries were checked for any possible keyboard error. Analysis was done in two-phase crossover trials for OGTT (Phase 1) and CGMS (Phase 2), separately. For both phases of the study, the period effect and period and treatment interaction were assessed while computing the treatment effect [16]. For both the phases, in each of the two groups, i.e., premeal almond load treatment vs. control; the number of participants analyzed was 60. For both phases, measurements taken repeatedly were converted into the area under the curve (AUC) which was analyzed and the primary outcome variable was postprandial blood glucose levels (AUC 0–2 h on OGTT and PP AUC 0–4 h on CGMS). AUC was calculated using the trapezoidal rule for all. The results are shown as AUC/time. For each participant, we had values of changes in parameters for premeal almond load treatment and standard OGTT. Therefore, paired *t*-test (change in premeal almond load test meal vs. standard meal) was used to compute the treatment effect (95% CI). STATA 15.0 statistical software (College Station, Texas, USA) was used for data analysis. In this study *p* value < 0.05 has been considered as statistically significant.

## Results

### Effect of premeal almond load treatment on post-75 g oral glucose load metabolic profile

A total of 60 participants (*n* = 30, each period) participated in this phase of the study (Control diet: only OGTT; treatment diet: diet with premeal almond load). Table 1 shows baseline and demographic characteristics. The overall AUC (0–2 h) (treatment: 1057.1 ± 101.7 vs. control: 1290.0 ± 134.0, *p* < 0.001) and AUC per unit time for blood glucose post-75 g oral glucose load was significantly lower for premeal almond load treatment diet as compared with control diet (*p* < 0.001). Mean blood glucose levels at 30, 60, 90, and 120 min were significantly lower for premeal almond load treatment diet vs. the control diet (*p* < 0.001 for 30, 60, 90, and 120 min). The AUC per unit time for serum insulin and mean serum insulin levels at 60, 90, and 120 min were significantly lower for premeal almond load treatment diet vs. the control diet (*p* < 0.001). The AUC per unit time for serum C-peptide (*p* < 0.01) and the mean values for serum C-peptide levels (*p* < 0.001 at 60 and 120 min) and overall AUC per unit time for plasma glucagon were significantly lower for participants on premeal almond load treatment diet as compared with those on control diet (*p* < 0.001). Levels of serum triglycerides, serum GLP-1, and plasma DPP-4 did not differ between the two diets (Table 2 and Fig. 2.1–2.7).

**Table 1:** Baseline characteristics.

Variables		*n*	%
Gender	Male	27	45
	Female	33	55
Age (years)	Mean ± SD	41.3 ± 7.9	
Family type	Joint	25	41.7
	Nuclear	27	45
	Extended	8	13.3
Monthly income (rupees)	<50,000	32	53.3
	>50,000	28	46.7
Consume tobacco	Not at all	52	86.7
	Sometimes	0	0
	Regularly	8	13.3
Consumption of alcohol	Not at all	39	65
	Sometimes	10	16.7
	Regularly	11	18.3
Body mass index (kg/m^2^)	Overweight	6	10
	Obese	54	90
Weight (kg)	Mean	79.2	
	Minimum, maximum	50.6, 116	
Resting pulse/min	Mean	78	
	Minimum, maximum	64, 104	
Systolic blood pressure (mmHg)	Mean	125.4	
	Minimum, maximum	90, 150	
Diastolic blood pressure (mmHg)	Mean	80.9	
	Minimum, maximum	56, 98

**Table 2:** Comparison of blood parameters computed from total area under the curve during 150 min between treatment and control diets.

Variables/group	Period 1 (mean ± SD)	Period 2 (mean ± SD)	Effect size (95% CI)
Blood glucose (mmol/L)			
Treatment	7.0 ± 0.7	6.8 ± 1.5	−1.6 (−1.8, −1.4) <0.001
Control	8.5 ± 1.6	8.4 ± 0.8	
Serum insulin (pmol/L)			
Treatment	412.2 ± 135.6	355.2 ± 142.2	−50.4 (−64.8, −36.6) <0.001
Control	406.2 ± 163.8	462.6 ± 136.8	
Plasma glucagon (pmol/L)			
Treatment	159.3 ± 69.9	148.8 ± 68.1	8.4 (−14.2, −2.7) <0.001
Control	157.3 ± 68.2	167.8 ± 72.1	
Serum C-peptide (nmol/L)			
Treatment	0.9 ± 0.3	1.0 ± 0.4	−0.3 (−0.3, −0.2) <0.001
Control	1.3 ± 0.5	1.1 ± 0.5	
Serum glucagon-like peptide-1 (pmol/L)^a^			
Treatment	3.9 ± 2.2	3.8 ± 2.4	−0.2 (−0.4,0.0) 0.089
Control	3.4 ± 2.2	3.7 ± 0.9	
Plasma dipeptidyl peptidase-4 (pg/mL)^a^			
Treatment	831.0 ± 1.6	1247.5 ± 3.5	0.1 (−0.2, 0.3) 0.579
Control	1333.9 ± 3.4	900.4 ± 4.8	
Serum triglycerides (mmol/L)			
Treatment	2.8 ± 0.7	3.3 ± 0.8	−0.01 (−0.1, 0.04) 0.945
Control	3.3 ± 0.8	2.8 ± 0.7	

^a^Data are represented in geometric mean and standard deviation (SD). In each of the groups (treatment and control), for each period number of subjects analyzed was *n* = 30. Treatment means the group that received a premeal almond load 30 min before the oral glucose tolerance test. Control means standard oral glucose tolerance test alone. Period 1 refers to the first order; Period 2 refers to the second order.

**Fig. 2:**
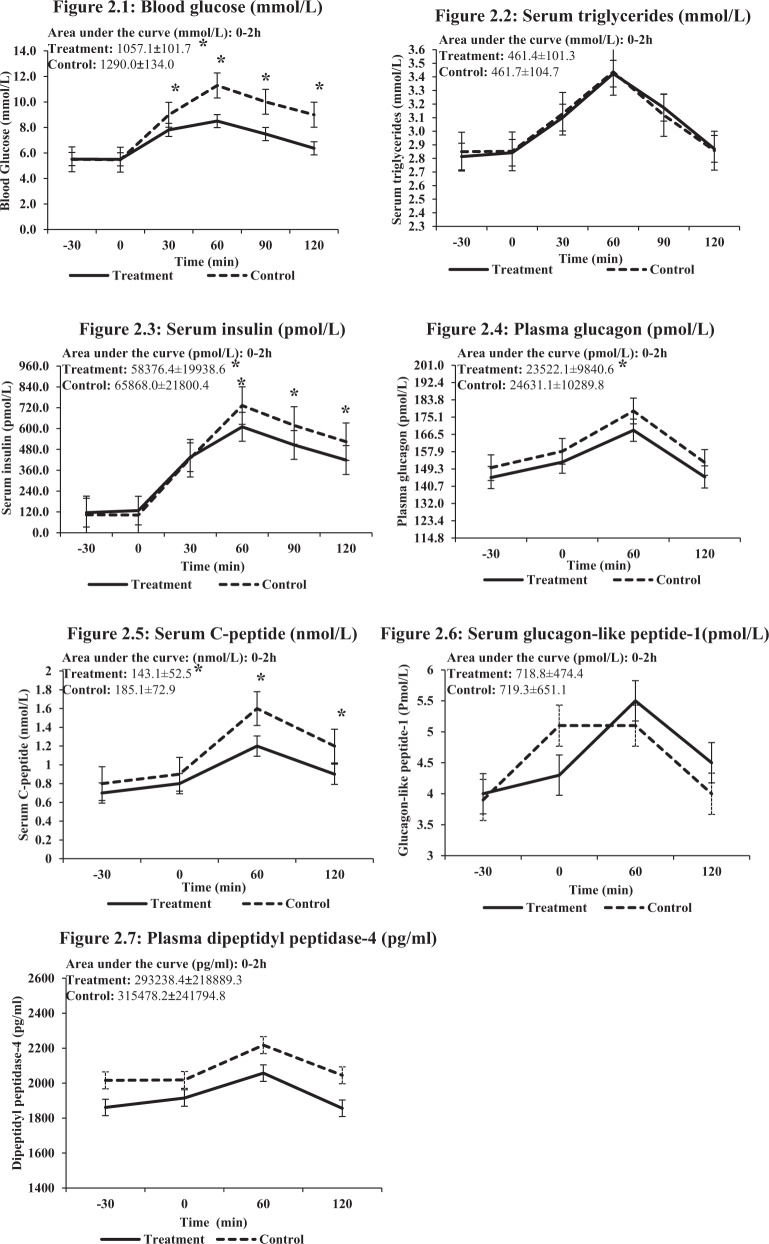
Mean values of glycemia and other metabolic parameters at −30, 0, 30, 60, 90, and 120 min (before and after 75 g oral glucose load). The solid line represents premeal almond load treatment and the dotted lines represent control diets. The X-axis shows time in min and Y-axis shows the values of the respective parameters. **2.1** Blood glucose (mmol/L). The X-axis shows the time points, and the Y-axis shows blood glucose values from 0 to 13.9 mmol/L. **2.2** Serum triglycerides (mmol/L). The X-axis shows the time points, and the Y-axis shows serum triglycerides values from 2.3 to 3.6 mmol/L. **2.3** Serum insulin (pmol/L). The X-axis shows the time points, and the Y-axis shows serum insulin values from 0 to 960 pmol/mL. **2.4** Plasma glucagon (pmol/L). The X-axis shows the time points, and the Y-axis shows plasma glucagon values from 114.8 to 201.0 pmol/L. **2.5** Serum C-peptide (nmol/L). The X-axis shows the time points, and the Y-axis shows serum c-peptide values from 0 to 2.0 nmol/L. **2.6** Serum glucagon-like peptide-1 (GLP-1) (pmol/L). The X-axis shows the time points, and the Y-axis shows GLP-1 values from 3 to 6 pmol/L. **2.7** Plasma dipeptidyl peptidase-4 (DPP-4) (pg/mL). The X-axis shows the time points, and the Y-axis shows plasma DPP-4 values from 1400 to 2600 pg/mL.

### Effect of premeal almond load on glucose profile using 3-day CGMS

**Fig. 3:**
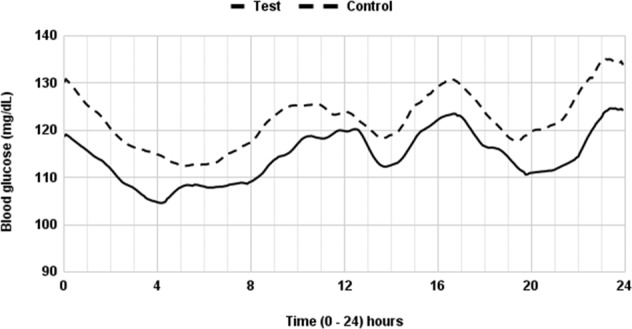
Continuous glucose monitoring showing the average blood glucose value of 60 participants. Note that average 72-h blood glucose levels are depicted in the 24-h graph. The solid line indicates premeal almond load treatment (n, 30) and the dotted line indicates the control diet (n, 30). The X-axis shows the time of continuous glucose monitoring over 24 h and the Y-axis shows blood glucose values from 90–140 mg/dL (5 to 7.8 mmol/L).

A series of analyses of the CGMS data show that almonds consistently induced greater improvement in circadian glucose control. Mean blood glucose profiles over the two CGM recording periods (of 3 complete days each) on premeal almond load treatment and control diets are shown in Fig. 3. A premeal almond load treatment significantly decreased mean daily glycaemia (M) in comparison to the control diet [−0.4 (−0.6, −0.3), *p* < 0.001] (Table 3). Time spent in the hyperglycaemic range over 7.8 mmol/L was lower in premeal almond load treatment diet vs. control diet [−0.04 (−0.1, −0.03), *p* < 0.001] and the corresponding AUC [–53.7 (−78.1–29.2), *p* < 0.001]. A significantly lower value for peak 24 h glucose [−0.8 (−12.8, −0.3), *p* < 0.001] was seen in the premeal almond load treatment diet, which was consistent with the significantly lower value for minimum blood glucose reading during the night [−0.4 (−0.7, −0.1), *p* < 0.001] Importantly, PPBG (AUC pp) also significantly decreased with premeal almond load treatment [−51.8 (96.6, −6.9), *p* < 0.02]. Overall, there was a significant decrease in glycaemic exposure (AUC total), [−43.3 (−67.4, −19.1), *p* < 0.001] in premeal almond load treatment vs. control diet.

**Table 3:** Circadian glycaemic control in premeal almond load treatment and control diet using continuous blood glucose monitoring system (CGM) measures.

Variable/group	Period 1	Period 2	Effect size (95% CI)
*Glycemic variability*			
Mean amplitude of glucose excursions (MAGE) (mmol/L)^a^			
Treatment	0.7 ± 0.1	0.8 ± 0.1	−0.1 (−0.1, 0.1) 0.060
Control	0.8 ± 0.1	1.1 ± 0.1	
Standard deviation of mean 24-h CGMS blood glucose readings (mmol/L)^a^			
Treatment	0.9 ± 0.1	1.0 ± 0.1	−0.1 (−0.1, −0.1) 0.020
Control	1.0 ± 0.1	1.1 ± 0.1	
Mean of daily differences (MoDD)			
Treatment	0.9 ± 0.1	1.1 ± 0.1	−0.1 (−0.1, −0.1) <0.001
Control	1.1 ± 0.1	1.2 ± 0.1	
*Glycemic control*			
Mean 24-h blood glucose reading (M) (mmol/L) mean (±SD)			
Treatment	6.2 ± 0.8	6.4 ± 0.7	−0.4 (−0.6, −0.3) <0.001
Control	6.6 ± 0.5	6.9 ± 0.8	
Time (min) spent in ideal range (3.9–7.8 mmol/L) (h) mean (±SD)			
Treatment	5.8 ± 1.5 × 10^1^	5.9 ± 1.5 × 10^1^	3.5 (−0.3, 7.3) 0.067
Control	5.8 ± 1.0 × 10^1^	5.3 ± 1.2 × 10^1^	
Area under of the curve (AUC) ideal range (3.9–7.8 mmol/h/L) mean (±SD)			
Treatment	35.1 ± 8.2 × 10^1^	36.6 ± 9.3 × 10^1^	8.4 (−14.7, 31.6) 0.469
Control	36.7 ± 6.4 × 10^1^	33.3 ± 6.4 × 10^1^	
Time spent above 3.9–7.8 mmol/L per day (h) mean (±SD)^a^			
Treatment	3.7 ± 0.1	5.0 ± 0.04	−0.04 (−0.1, −0.03) <0.001
Control	9.1 ± 0.04	12.3 ± 0.04	
AUC >7.8 mmol/L per day (mmol/h/L) mean (±SD)			
Treatment	5.8 ± 1.1 × 10^1^	6.8 ± 7.7 × 10^1^	−53.7 (−78.1–29.2) <0.001
Control	8.9 ± 6.3 × 10^1^	14.4 ± 1.1 × 10^1^	
Peak 24-h glycaemia reading (mmol/L) mean (±SD)			
Treatment	8.4 ± 1.5	8.9 ± 2.3	−0.8 (−12.8, −0.3) <0.001
Control	9.0 ± 1.1	9.9 ± 1.6	
Minimum blood glucose reading during the night (mmol/L) mean (±SD)			
Treatment	4.9 ± 0.8	5.1 ± 0.9	−0.4 (−0.7, −0.1) <0.001
Control	5.3 ± 0.7	5.4 ± 0.7	
Postprandial hyperglycemia AUC pp (mmol/h/L) mean (±SD)			
Treatment	40.7 ± 11.4 × 10^1^	45.4 ± 16.5 × 10^1^	−51.8 (96.6, −6.9) 0.020
Control	47.5 ± 20.2 × 10^1^	48.9 ± 14.9 × 10^1^	
Overall hyperglycemia AUC total (mmol/h/L) mean (±SD)			
Treatment	41.7 ± 8.5 × 10^1^	44.1 ± 8.8 × 10^1^	−43.3 (−67.4, −19.1) <0.001
Control	46.4 ± 5.3 × 10^1^	48.0 ± 6.7 × 10^1^	
Basal hyperglycemia AUC (mmol/h/L) mean (±SD)			
Treatment	32.8 ± 11.1	35.7 ± 10.1	0.7 (−3.4, 4.8) 0.727
Control	33.1 ± 11.6	33.9 ± 13.6	

^a^Data are represented in geometric mean and standard deviation (SD). In each of the groups (treatment and control), for each period, the number of subjects analyzed was *n* = 30. Treatment means the group that received premeal almond load 30 min before the oral glucose tolerance test. Control means standard oral glucose tolerance test alone. Period 1 refers to first order; Period 2 refers to second order. AUC_PP is calculated per meal for 4 h after the meal.

*CGMS* continuous glucose monitoring system.

Analysis of 24-h variability as reflected by the MAGE Index shows a statistically non-significant difference between premeal almond load treatment and control diets. A significantly lower value of the standard deviation of mean glucose concentration [−0.1 (−0.1, −0.1), *p* < 0.02] and MoDD with premeal almond load treatment [−0.1 (−0.1, −0.1), *p* < 0.001] was observed vs. control diet (Table 3 and Fig. 3). Since MAGE and Standard deviation of mean glucose concentration are not affected by basal levels of blood glucose. A decrease in these values may mean a more robust benefit.

CGMS report of one of the best performers (a 41-year-old male with prediabetes) has been shown in Supplementary Fig. 1. It is evident that when the subject was consuming almonds as a preload, the PPBG spikes were lower in comparison to when the subject was on the control diet. Furthermore, lower blood glucose excursions throughout the day with premeal almond load treatment vs. controls were seen, particularly in the postprandial period (Supplementary Fig. 1.1 and 1.2).

## Discussion

This is the first study to investigate the effects of a premeal almond load on parameters of glycemia in Asian Indians with prediabetes. As mentioned previously, there is only one study by Crouch et al. [13] in which the effect of premeal almond load treatment on blood glucose levels was studied. Furthermore, an assessment of multiple parameters of glycemia with CGMS after preloading of almonds has been done for the first time. In our study, ingestion of 20 g of almonds 30 min before an oral glucose load showed a significant decrease in blood glucose, serum insulin, plasma glucagon, and serum C-peptide in the premeal almond load treatment diet as compared to the control diet (only OGTT) in OGTT-based study. We also observed an overall lower AUC for blood glucose, serum insulin, serum C-peptide, and plasma glucagon in participants on the premeal almond load treatment diet as compared to those on the control diet. Specifically, premeal almond load treatment decreases PPBG (AUC) by 18.05%, and 10.07% than the control diet on OGTT and CGMS, respectively.

It is important to note that the prevalence of prediabetes is rising in India [17] and so is the prevalence of CVD [18]. As discussed in the introduction, prediabetes is an important risk factor for T2D and CVD [19]. Daily glucose excursion adversely affects coronary plaque vulnerability in participants with CVD and those pre-treated with lipid-lowering therapy [20]. Further important observations in this study included that a large glucose fluctuation is the only independent risk factor for the progression of the necrotic core within the coronary plaque and the formation of thin-cap fibroatheroma. Specifically, 2-h post OGTT blood glucose value is strongly associated with mortality and CVD [21]. The DECODE (Diabetes Epidemiology: Collaborative Analysis of Diagnostic Criteria in Europe) study showed that high blood glucose concentrations 2 h after OGTT were associated with an increased risk of death, independent of fasting blood glucose [22]. The mechanism by which blood glucose excursion increases CVD risk may be the induction of inflammation, oxidative stress, and/or endothelial dysfunction. PPBG was shown to contribute to the progression of coronary artery disease (CAD) in Japanese participants with angina [23]. This study also showed that participants with 2-h post-challenge glucose levels greater than 7.8 mmol/L had higher CAD severity scores.

Almonds and other nutrients have been given at various times in relation to major meals to reduce blood glucose levels. Several investigators have shown that almonds given with meals lower PPBG. In studies that evaluated meals with almonds matched for carbohydrate content, PPBG concentrations were reduced by 9–20% in healthy individuals, and 7–30% in individuals with prediabetes and T2D [24–29]. Josse et al. [25] (*n* = 9, healthy participants) and Jenkins et al. [24] (*n* = 15, healthy participants) showed a 21–42% reduction in peak PPBG by adding 30–90 g of almonds to a test meal containing 50 g of carbohydrates. Cohen et al. [28] (*n* = 13, healthy participants, 7 participants with T2D), reported that a standard serving of almonds given along with meals significantly reduced PPBG in participants with diabetes (−30%, *p* = 0.043) but did not influence glycemia in participants without diabetes (−7%, *p* = 0.638). Tan and Mattes [29] (*n* = = 137, individuals at high risk for developing diabetes) showed that 43 g (1.5 oz) of almonds given as midmorning and mid-afternoon snacks lowered postprandial blood glucose.

In our study change in glucose AUC is greater than the insulin AUC for premeal almond load. OGTT graph (Fig. 2.3) shows a slight deceleration of insulin increment between 30 and 60 min after which the lines of the graph are parallel. While this may mean a slight lagging behind of insulin secretion because of an overall significant decrease in AUC insulin. More importantly, this means a lower magnitude of hyperinsulinemia. Such a decrease in blood glucose and a decrease in hyperinsulinemia will likely benefit glucose-insulin metabolism in the long run. A decrease in fasting and postprandial insulin has been shown recently by us in a paper, where pharmacological intervention (dapagliflozin) has been done in patients with T2D for a period of 120 days [30].

Studies in which other nutrients (e.g., fructose and soya yogurt) were used as premeal almond load also showed encouraging results. Heacock et al. [31] reported that ingestion of 10 g of fructose 30 or 60 min before eating a high-carbohydrate test meal reduced the incremental area under the glucose curve by 25–27% in 32 healthy participants. In 10 participants with T2D treated either by diet/metformin, a soya yogurt snack (30 g of soybeans and 75 g of yogurt) 2 h before breakfast reduced the increment of PPBG by 40% [32]. The magnitude of AUC reduction on OGTT was higher (18.05%) in our study than that observed by Crouch et al. (15.5%) [13] but less than the other studies in which fructose or soya yogurt preload was used [31, 32]. Crouch et al. [13] reported a mean difference between standard OGTT and OGTT after premeal almond load at 1 and 2 h as 19.4% and 14.1%, respectively, whereas we observed a greater reduction in blood glucose values at 1 h (24.8%) at 2 h (28.9%) on premeal almond load treatment diet. It is to be noted that while we used OGTT to test this, other investigators have used test meals or meals along with nutrients. In participants with prediabetes, such reduction in glycemia may have important implications for the reversal of diabetes as well.

Besides HbA1C, the role of glycaemic variability in relation to the development of complications of diabetes is increasingly being researched. Glycaemic variability is denoted by intraday glycaemic excursions, including episodes of hyperglycemia and hypoglycemic. These short-term changes, frequent glucose fluctuations, may independently contribute to diabetes-related complications [33–35]. One of the factors by which the effectiveness of anti-hyperglycaemic therapies (DPP-4 inhibitors, GLP-1-based therapies, newer insulins, insulin pumps, and bariatric surgery) is how much these modalities of treatment can decrease glycaemic variability. In the current study, we observed a significant difference in parameters of glycaemic variability between two diets (standard deviation of mean 24-h CGMS blood glucose, MoDD). Although glycaemic variability is not pronounced in individuals with prediabetes because blood glucose fluctuations occur within a narrower range than in participants with type 1 diabetes and T2D, yet we have observed a significant effect of premeal almond load treatment on parameters of glycaemic variability. It would also be worthwhile to include almonds in the diets of participants with T2D, where effects on glycaemic variability may be more pronounced.

It is difficult to comment on the exact mechanism of action of preloading almonds. The following have been researched and discussed. (a) Premeal almond load stimulates the release of stored insulin 30 min sooner than insulin release stimulated by the 75-g oral glucose load. This hypothesis is described as priming the “pancreatic pump” [13]. (b) Fiber content of almonds increases the viscosity of intestinal contents which hinders glucose diffusion. Furthermore, its fat content may slow gastric emptying time, thus delaying glucose absorption [29]. (c) Almonds have high zinc and magnesium contents that could stimulate the tyrosine kinase receptor in adipose tissues, thus improving insulin sensitivity [36]. (d) The high content of monounsaturated fatty acids (MUFAs) in almonds may also increase insulin sensitivity. (e) A reduction in hunger [37]. These mechanisms may be operative in combination to reduce PPHG.

Individuals with prediabetes and T2D demonstrate lower GLP-1 response to an oral glucose load in comparison to healthy individuals [38]. Interestingly, prebiotic properties of almonds may result in increased GLP-1 levels as shown in an in vitro study [39]. In addition, almond supplementation, through its action on *Bifidobacterium* and *Lactobacillus* (which increase the production of short-chain fatty acids) [40] may increase GLP-1 levels [38, 39]. Mori et al. [26] did not find any increase in GLP-1 concentrations with almond ingestion, but it increased after increased mastication of almonds [41]. Cohen et al. [28] showed when almonds are consumed along with meals, insulinemia and GLP-1 levels at 30 min post-meal were not impacted by almond ingestion for either diet. In the current study, we did not observe any significant effect on GLP-1 levels with the premeal almond load treatment.

It is known that serum C-peptide levels parallel the insulin secretory capacity of pancreatic beta cells. The levels would increase in those having insulin resistance and hyperinsulinemia. Jenkins et al. [42] observed lower 24-h urinary C-peptide output after creatinine correction with almond consumption compared with the control treatment. Chen et al. [43] found no effect of almond consumption on C-peptide levels compared with the control treatment. In a study by Rock et al. [44] significantly lower values of C-peptide levels at 120 min were noted after consuming a walnut-containing meal. In parallel with the results of these multiple studies, significantly lower C-peptide values at 60 and 120 min post ingestion of 75 g of glucose were shown in our study with premeal almond load treatment as compared to controls.

Effects of almond consumption on plasma glucagon and plasma DPP-4 levels have not been evaluated previously. Importantly, high circulating DPP-4 levels are linked to several adverse effects including those on the liver, and such high levels are prevalent in Asian Indian participants with T2D [45]. Glucagon levels are important for blood glucose physiology. High glucagon levels increase hepatic glucose output and contribute to hyperglycemia [46]. Interestingly, we have previously shown high glucagon levels in newly diagnosed Asian Indian patients with T2D vs. control [47]. We now know how to decrease glucagon levels with drugs (DPP-4 inhibitors) but it is a fact that nutrient manipulation needs to be researched. A study by Rock et al. [44] showed that walnut ingestion led to higher glucagon values than the reference meal at 120 min. These findings contrast with our study, which shows significantly lower overall AUC for glucagon in subjects on premeal almond load treatment diet. Again, our findings are important because lowering glucagon is one of the important targets of current antihyperglycemic therapy (e.g., DPP-4 inhibitors).

## Conclusion

The present study shows how the adoption of a simple change to an individual diet, namely the incorporation of 20 g of almonds, 30 min before each major meal (breakfast, lunch, and dinner), leads to a significant decrease in PPBG, insulin, C-peptide levels, glucagon, on OGTT and improved multiple measures of glycaemic variability and glycaemic control on CGMS. Adopting such strategies to manage hyperglycemia, especially in a prediabetes state can be helpful in delaying or controlling conversion to diabetes or even help reversal to normal glucose regulation. This is even more important in the case of the Asian Indian population, where the prevalence of diabetes is increasing at an alarming pace every year, and PPHG is a prevalent abnormality in the blood glucose profile. The metabolic benefits of premeal almond load treatment as seen in our study in participants with prediabetes are generalizable to a substantial number of people with prediabetes in India. As discussed previously, it has major implications for better glycaemic control in participants with diabetes as well.

## Supplementary information

Supplementary Table 1: Nutrition information of 20 and 60 g of almonds

Supplementary File 1

Supplementary Table 2: Meal-wise distribution of treatment diet and control diet

Supplementary Table 3: The macronutrient distribution of treatment and control diet

Supplementary Figure 1

## Data Availability

All relevant raw data will be freely available to any researcher wishing to use them for non-commercial purposes, without breaching participant confidentiality.
