# Supplementary material for: Beneficial effects of premeal almond load on glucose profile on oral glucose tolerance and continuous glucose monitoring: randomized crossover trials in Asian Indians with prediabetes
Source: Eur J Clin Nutr. 2023 Feb 2;77(5):586–95. doi: 10.1038/s41430-023-01263-1 (PMC10169634; doi:10.1038/s41430-023-01263-1)
Supplement: Supplementary file 1 — Supplementary Table 1: Nutrition information of 20 and 60 g of almonds [file 41430_2023_1263_MOESM1_ESM.docx]

**Supplementary Table1: Nutrition information of 20 and 60 g of almonds**

| **Almond** | **Energy** | **Protein** | **Fat** | **Carbohydrate** | **Fiber** |
| --- | --- | --- | --- | --- | --- |
| 20gm | 114.3 | 4.3 | 10 | 4.3 | 2.9 |
| 60gm | 342.9 | 12.9 | 30 | 12.9 | 8.7 |
