# Supplementary material for: Beneficial effects of premeal almond load on glucose profile on oral glucose tolerance and continuous glucose monitoring: randomized crossover trials in Asian Indians with prediabetes
Source: Eur J Clin Nutr. 2023 Feb 2;77(5):586–95. doi: 10.1038/s41430-023-01263-1 (PMC10169634; doi:10.1038/s41430-023-01263-1)
Supplement: Supplementary file 2 — Supplementary File 1 [file 41430_2023_1263_MOESM2_ESM.docx]

Supplementary file 1

**Blood Glucose (mg/dl)**

*

**Area under the Curve:**

**Treatment:** 19028.1±1830.1

**Control:**23219.8±2411.1

**Serum Insulin(uU/ml)**

*

**Area under the Curve:**

**Treatment:** 9729.4±3323.1

**Control:**10978.0±3633.4

**Area under the Curve:**

**Treatment:** 293238.4±218889.3

**Control:**315478.2±241794.8

**Area under the Curve:**

**Treatment:** 432.3±158.7

**Control:**559.2±220.2

*

1. MAGE

Mean Amplitude of Glucose Excursion was calculated using a software algorithm which would identify all the Glucose peaks and nadirs from a baseline cut off value. The absolute difference of glucose peaks and nadirs from the baseline is calculated and averaged to get MAGE.


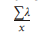

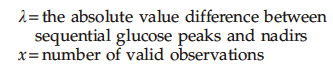


1. MODD:

The “Mean of Daily Differences” is a measure of the day-to-day variation of the glucose pattern. It’s defined as the mean of the absolute differences between glucose values on day 2 and the corresponding values on day 1, at the same time.

1. [Area Under the Curve (AUC)](javascript:HMToggle('toggle','TOGGLE0186A3'))

The “Glucose Area Above the Target Limit” is determined by calculating the area (glucose concentration * time) “mg/dL * min.” created by the sensor tracing when it exceeds the patient upper target range.

The “Glucose Area Below the Target Limit” is determined by calculating the area (glucose concentration * time) “mg/dL *min.” created by the sensor tracing when it is below the patient lower target range.

Formula to calculate AUC, normalized to 24 hours for each day

$(\sum\left| SG Value - Limit \right|*5 min {}/{(Number of SG Values)}$) * 288

1. Basal Hyperglycemia (AUC_B):

SG Values above Limit between 0 to 6am or to the first meal marker, whichever earlier, are included in the calculation.

Formula to calculate AUC_B

$((\sum|SG Value - Limit| )*5 min$)

1. Overall hyperglycemia:

SG Values above Limit for entire time of data collection is included in the calculation.

Formula to calculate overall hyperglycemia

$((\sum|SG Value - Limit| )*5 min$)

1. Minimum blood glucose reading during night

The lowest SG Values between 0 to 6am is calculated to get the minimum blood glucose reading at night.

$$MIN(SG Values at night)$$

1. Peak 24 hyperglycemia

The highest SG Values in a given 24 hours is calculated to get the peak 24 Hyperglycemia.

$$MAX(SG Values in 24 hours)$$

1. Time above 200

For a given time period, whether 24 hours or several days, number of SG values above 200 mg/dL are calculated. This value is the multiplied by the frequency of SG value collection to get this value.

$$(number of observations)*5 min$$

1. AUC more than 140

For a given time period, whether 24 hours or several days, observations when SG values above 140 mg/dL are identified. The SG values are summed for the observations and multiplied by the frequency of SG value collection.

$$(\sum SG Value when above 140 mg/dL )*5 min$$

1. Time spent above 140

For a given time period, whether 24 hours or several days, observations when SG values above 140 mg/dL are identified. This value is the multiplied by the frequency of SG value collection to get this value.

$$(number of observations)*5 min$$

1. AUC ideal range

For a given time period, whether 24 hours or several days, observations when SG values within ideal range are identified. The SG values are summed for the observations and multiplied by the frequency of SG value collection.

$$(\sum SG Value when in ideal limit )*5 min$$

1. Time spent in ideal range

For a given time period, whether 24 hours or several days, observations when SG values within ideal range are identified. This value is the multiplied by the frequency of SG value collection to get this value.

$$(number of observations)*5 min$$

1. Mean 24 hr. blood glucose reading.

For a given 24 hours of SG data, all the SG values are summed and divided by the total number of SG data points to calculated the mean 24 hours blood glucose reading.

$$(\sum SG Value in a given 24 hours )/288$$
