# Supplementary material for: Beneficial effects of premeal almond load on glucose profile on oral glucose tolerance and continuous glucose monitoring: randomized crossover trials in Asian Indians with prediabetes
Source: Eur J Clin Nutr. 2023 Feb 2;77(5):586–95. doi: 10.1038/s41430-023-01263-1 (PMC10169634; doi:10.1038/s41430-023-01263-1)
Supplement: Supplementary file 3 — Supplementary Table 2: Meal-wise distribution of treatment diet and control diet [file 41430_2023_1263_MOESM3_ESM.docx]

Supplementary Table 2: Meal wise distribution of treatment diet and control diet

| Treatment diet (n=30) | | | | | | |
| --- | --- | --- | --- | --- | --- | --- |
| 24-hour meal composition | Energy | | Carbohydrate | Protein | Fat | |
| Cereal (whole wheat flour) 125g | 426.3 ± 30.7 | | 86.8 ± 15.3 | 15.1 ± 1.3 | 2.1 ± 0.3 | |
| Lentils (45g) | 154.4 ± 12.3 | | 28.4 ± 6.7 | 9 ± 0.8 | 0.8 ± 0.1 | |
| Visible fat (cooking oil) 7.5g | 67.5 ± 6.8 | | - | - | 7.5 ± 0.5 | |
| Vegetable (450g) | 63 ± 6.3 | | 12 ± 1.1 | 1 ± 0.1 | 0.8 ± 0.1 | |
| Fat free dairy (curd) 75g | 120 ± 11.2 | | 3 ± 0.4 | 20 ± 1.3 | 1.7 ± 0.3 | |
| Buttermilk (250ml) | 37.5 ± 3.4 | | 1.3 ± 0.1 | 2 ± 0.3 | 3.5 ± 0.4 | |
| 60g almond | 342.9 ± 21.5 | | 12.9 ± 2.3 | 12.9 ± 2.1 | 30 ± 6.4 | |
| Salad (300g) | 86 ± 7.1 | | 16 ± 1.7 | 3 ± 0.6 | 0.6 ± 0.1 | |
| Total (g) | 1297.5 ± 99.3 | | 160.3 ± 27.6 | 63 ± 6.5 | 46.9 ± 8.2 | |
| Total (%) |  | | 49.4 | 19.4 | 32.5 | |
| Breakfast | | | | | | |
| 20g almond | 114.3 ± 7.5 | | 4.3 ± 0.7 | 4.3 ± 0.6 | | 10 ± 1.9 |
| Cereal (whole wheat flour) 50g | 170.5 ± 9.4 | | 34.7 ± 6.0 | 6 ± 0.8 | | 0.8 ± 0.1 |
| Vegetable (150g) | 21 ± 2.3 | | 4 ± 0.5 | 0.3 ± 0.1 | | 0.3 ± 0.1 |
| Visible fat (cooking oil) 1.5g | 13.5 ± 1.4 | |  |  | | 1.5 ± 0.3 |
| Salad (150g) | 43 ± 3.7 | | 8 ± 0.8 | 1.5 ± 0.3 | | 0.3 ± 0.1 |
| Total (g) | 362.3 ± 24.3 | | 51 ± 7.0 | 12.2 ± 1.8 | | 12.9 ± 2.5 |
| Total (%) |  | | 56.3 | 13.4 | | 32 |
| Lunch | | | | | | |
| 20g almond | 114.3 ± 7.1 | 4.3 ± 0.7 | | 4.3 ± 0.7 | | 10 ± 2.1 |
| Cereal (whole wheat flour) 40g | 136.4 ± 7.4 | 27.8 ± 4.5 | | 4.8 ± 0.5 | | 0.7 ± 0.1 |
| Lentils (22.5g) | 77.2 ± 6.1 | 14.2 ± 3.2 | | 4.5 ± 0.4 | | 0.4 ± 0.1 |
| Vegetable (150g) | 21 ± 1.9 | 4 ± 0.5 | | 0.3 ± 0.1 | | 0.3 ± 0.1 |
| Fat free dairy (curd) 75g | 120 ± 11.9 | 3 ± 0.3 | | 20 ± 1.2 | | 1.7 ± 0.2 |
| Visible fat (cooking oil) 3g | 27 ± 2.7 |  | |  | | 3 ± 0.5 |
| Total (g) | 495.9 ± 37.1 | 53.2 ± 9.2 | | 33.9 ± 2.9 | | 16 ± 3.1 |
| Total (%) |  | 42.9 | | 27.3 | | 29 |
| Evening snack | | | | | | |
| Buttermilk (250ml) | 37.5 ± 3.3 | 1.3 ± 0.1 | | 2 ± 0.3 | | 3.5 ± 0.3 |
| Salad (150g) | 43 ± 3.5 | 8 ± 0.7 | | 1.5 ± 0.2 | | 0.3 ± 0.1 |
| Total (g) | 80.5 ± 6.8 | 9.3 ± 0.8 | | 3.5 ± 0.5 | | 3.8 ± 0.5 |
| Total (%) |  | 45.9 | | 17.4 | | 42.5 |
| Dinner | | | | | | |
| 20g almond | 114.3 ± 8.1 | 4.3 ± 0.8 | | 4.3 ± 0.7 | | 10 ± 2.2 |
| Cereal (whole wheat flour) 35g | 119.4 ± 7.4 | 24.3 ± 4.2 | | 4.2 ± 0.3 | | 0.6 ± 0.1 |
| Lentils (22.5g) | 77.2 ± 6.7 | 14.2 ± 3.1 | | 4.5 ± 0.3 | | 0.4 ± 0.1 |
| Vegetable (150g) | 21 ± 1.8 | 4 ± 0.4 | | 0.3 ± 0.1 | | 0.3 ± 0.1 |

| Visible fat (cooking oil) 3g | 27 ± 2.9 |  |  | 3 ± 0.5 |
| --- | --- | --- | --- | --- |
| Total (g) | 358.8 ± 26.9 | 46.8 ± 8.5 | 13.4 ± 1.4 | 14.2 ± 3.0 |
| Total (%) |  | 52.1 | 14.9 | 35.6 |
| Control diet | | | | |
| Cereal (whole wheat flour) 125g | 426.3 ± 18.4 | 86.8 ± 3.9 | 15.1 ± 1.7 | 2.1 ± 0.4 |
| Lentils (45g) | 154.4 ± 6.1 | 28.4 ± 1.2 | 9 ± 1.0 | 0.8 ± 0.1 |
| Visible fat (cooking oil) 40g | 360 ± 16.2 | - | - | 40 ± 5.4 |
| Vegetable (450g) | 63 ± 2.5 | 12 ± 0.7 | 1 ± 0.1 | 0.8 ± 0.1 |
| Fruit (100g) | 59 ± 2.6 | 13.4 ± 0.9 | 0.2 ± 0.1 | 0.8 ± 0.1 |
| Fat free dairy (curd) 75g | 120 ± 5.1 | 3 ± 0.3 | 20 ± 2.1 | 1.7 ± 0.4 |
| Buttermilk (250ml) | 37.5 ± 2.4 | 1.3 ± 0.2 | 2 ± 0.3 | 3.5 ± 0.5 |
| Salad (300g) | 86 ± 3.1 | 16 ± 1.0 | 3 ± 0.4 | 0.6 ± 0.1 |
| Total (g) | 1306.1 ± 56.2 | 160.8 ± 8.1 | 50.3 ± 5.8 | 50.3 ± 7.0 |
| Total (%) |  | 49.2 | 15.4 | 34.6 |
| Breakfast | | | | |
| Cereal (whole wheat flour) 50g | 170.5 ± 5.7 | 34.7 ± 1.1 | 6 ± 0.6 | 0.8 ± 0.1 |
| Vegetable (150g) | 21 ± 0.9 | 4 ± 0.3 | 0.3 ± 0.1 | 0.3 ± 0.1 |
| Visible fat (cooking oil) 12.5g | 112.5 ± 5.6 |  |  | 12.5 ± 1.8 |
| Apple (100g) | 59 ± 2.7 | 13.4 ± 1.0 | 0.2 ± 0.1 | 0.8 ± 0.1 |
| Fat free dairy (curd) 20g | 32 ± 0.6 | 0.8 ± 0.2 | 5.3 ± 0.6 | 0.5 ± 0.1 |
| Total (g) | 395 ± 15.7 | 52.9 ± 2.6 | 11.9 ± 1.4 | 14.9 ± 2.2 |
| Total (%) |  | 53.6 | 12.05 | 33.9 |
| Lunch | | | | |
| Cereal (whole wheat flour) 40g | 136.4 ± 5.2 | 27.8 ± 1.0 | 4.8 ± 0.5 | 0.7 ± 0.1 |
| Lentils (22.5g) | 77.2 ± 3.1 | 14.2 ± 0.7 | 4.5 ± 0.6 | 0.4 ± 0.1 |
| Vegetable (150g) | 21 ± 1.1 | 4 ± 0.4 | 0.3 ± 0.1 | 0.3 ± 0.1 |
| Visible fat (cooking oil) 12.5g | 112.5 ± 6.1 |  |  | 12.5 ± 2.0 |
| Fat free dairy (curd) 55g | 88 ± 1.7 | 2.2 ± 0.7 | 14.6 ± 1.7 | 1.3 ± 0.3 |
| Total (g) | 435.1 ± 17.3 | 48.1 ± 2.8 | 24.3 ± 2.9 | 15.1 ± 2.6 |
| Total (%) |  | 44.3 | 22.3 | 31.2 |
| Evening snack | | | | |
| Buttermilk (250ml) | 37.5 ± 2.3 | 1.3 ±0.3 | 2 ± 0.4 | 3.5 ± 0.6 |
| Salad (150g) | 43 ± 1.6 | 8 ± 0.5 | 1.5 ± 0.2 | 0.3 ± 0.1 |
| Total (g) | 80.5 ± 3.9 | 9.3 ± 0.8 | 3.5 ± 0.6 | 3.8 ± 0.7 |
| Total (%) |  | 45.9 | 17.4 | 42.5 |
| Dinner |  |  |  |  |
| Cereal (whole wheat flour) 35g | 119.4 ± 4.9 | 24.3 ± 0.9 | 4.2 ± 0.4 | 0.6 ± 0.1 |
| Lentils (22.5g) | 77.2 ± 3.0 | 14.2 ± 0.8 | 4.5 ± 0.5 | 0.4 ± 0.1 |
| Vegetable (150g) | 21 ± 1.0 | 4 ± 0.5 | 0.3 ± 0.1 | 0.3 ± 0.1 |
| Visible fat (cooking oil) 15g | 135 ± 6.0 |  |  | 15 ± 2.1 |

| Salad (150g) | 43 ± 1.7 | 8 ± 0.4 | 1.5 ± 0.3 | 0.3 ± 0.1 |
| --- | --- | --- | --- | --- |
| Total (g) | 395.5 ± 16.8 | 50.5 ± 2.6 | 10.6 ± 1.3 | 16.5 ± 2.5 |
| Total (%) |  | 51 | 10.7 | 37.6 |
