# Supplementary material for: Beneficial effects of premeal almond load on glucose profile on oral glucose tolerance and continuous glucose monitoring: randomized crossover trials in Asian Indians with prediabetes
Source: Eur J Clin Nutr. 2023 Feb 2;77(5):586–95. doi: 10.1038/s41430-023-01263-1 (PMC10169634; doi:10.1038/s41430-023-01263-1)
Supplement: Supplementary file 4 — Supplementary Table 3: The macronutrient distribution of treatment and control diet [file 41430_2023_1263_MOESM4_ESM.docx]

Supplementary Table 3: The macronutrient distribution of treatment and control diet

|  | Treatment diet (premeal load of almonds) | Control diet |
| --- | --- | --- |
| 24 Hour meal composition | | |
| Energy (kcal) | 1297.5 ± 99.3 | 1306.1 ± 56.2 |
| Carbohydrate (g) | 160.3 ± 27.6 | 160.8 ± 8.1 |
| Carbohydrate (%) | 49.4 | 49.23 |
| Protein (g) | 63.0 ± 6.5 | 50.3 ± 5.8 |
| Protein (%) | 19.4 | 15.4 |
| Fats (g) | 46.9 ± 8.2 | 50.3 ± 7.0 |
| Fats (%) | 32.5 | 34.63 |
| Meal wise composition | | |
| Breakfast | | |
| Energy (kcal) | 362.3 ± 24.3 | 395 ± 15.7 |
| Carbohydrate (g) | 51 ± 7.0 | 52.9 ± 2.6 |
| Carbohydrate (%) | 56.3 | 53.6 |
| Protein (g) | 12.2 ± 1.8 | 11.9 ± 1.4 |
| Protein (%) | 13.4 | 12.1 |
| Fats (g) | 12.9 ± 2.5 | 14.9 ± 2.2 |
| Fats (%) | 32 | 33.9 |
| Lunch | | |
| Energy (kcal) | 495.9 ± 37.1 | 435.1 ± 17.3 |
| Carbohydrate (g) | 53.2 ± 9.2 | 48.1 ± 2.8 |
| Carbohydrate (%) | 42.9 | 44.3 |
| Protein (g) | 33.9 ± 2.9 | 24.3 ± 2.9 |
| Protein (%) | 27.3 | 22.3 |
| Fats (g) | 16 ± 3.1 | 15.1 ± 2.6 |
| Fats (%) | 29 | 31.2 |
| Evening snack: | | |
| Energy (kcal) | 80.5 ± 6.8 | 80.5 ± 3.9 |
| Carbohydrate (g) | 9.3 ± 0.8 | 9.3 ± 0.8 |
| Carbohydrate (%) | 45.9 | 45.9 |
| Protein (g) | 3.5 ± 0.5 | 3.5 ± 0.6 |
| Protein (%) | 17.4 | 17.4 |
| Fats (g) | 3.8 ± 0.5 | 3.8 ± 0.7 |
| Fats (%) | 42.5 | 42.5 |

| Dinner | | |
| --- | --- | --- |
| Energy (kcal) | 358.8 ± 26.9 | 395.5 ± 16.8 |
| Carbohydrate (g) | 46.8 ± 8.5 | 50.5 ± 2.6 |
| Carbohydrate (%) | 52.1 | 51 |
| Protein (g) | 13.3 ± 1.4 | 10.6 ± 1.3 |
| Protein (%) | 14.9 | 10.7 |
| Fats (g) | 14.2 ± 3.0 | 16.5 ± 2.5 |
| Fats (%) | 35.6 | 37.5 |
