# Supplementary material for: Beneficial effects of premeal almond load on glucose profile on oral glucose tolerance and continuous glucose monitoring: randomized crossover trials in Asian Indians with prediabetes
Source: Eur J Clin Nutr. 2023 Feb 2;77(5):586–95. doi: 10.1038/s41430-023-01263-1 (PMC10169634; doi:10.1038/s41430-023-01263-1)
Supplement: Supplementary file 5 — Supplementary Figure 1 [file 41430_2023_1263_MOESM5_ESM.pptx]

## Slide 1
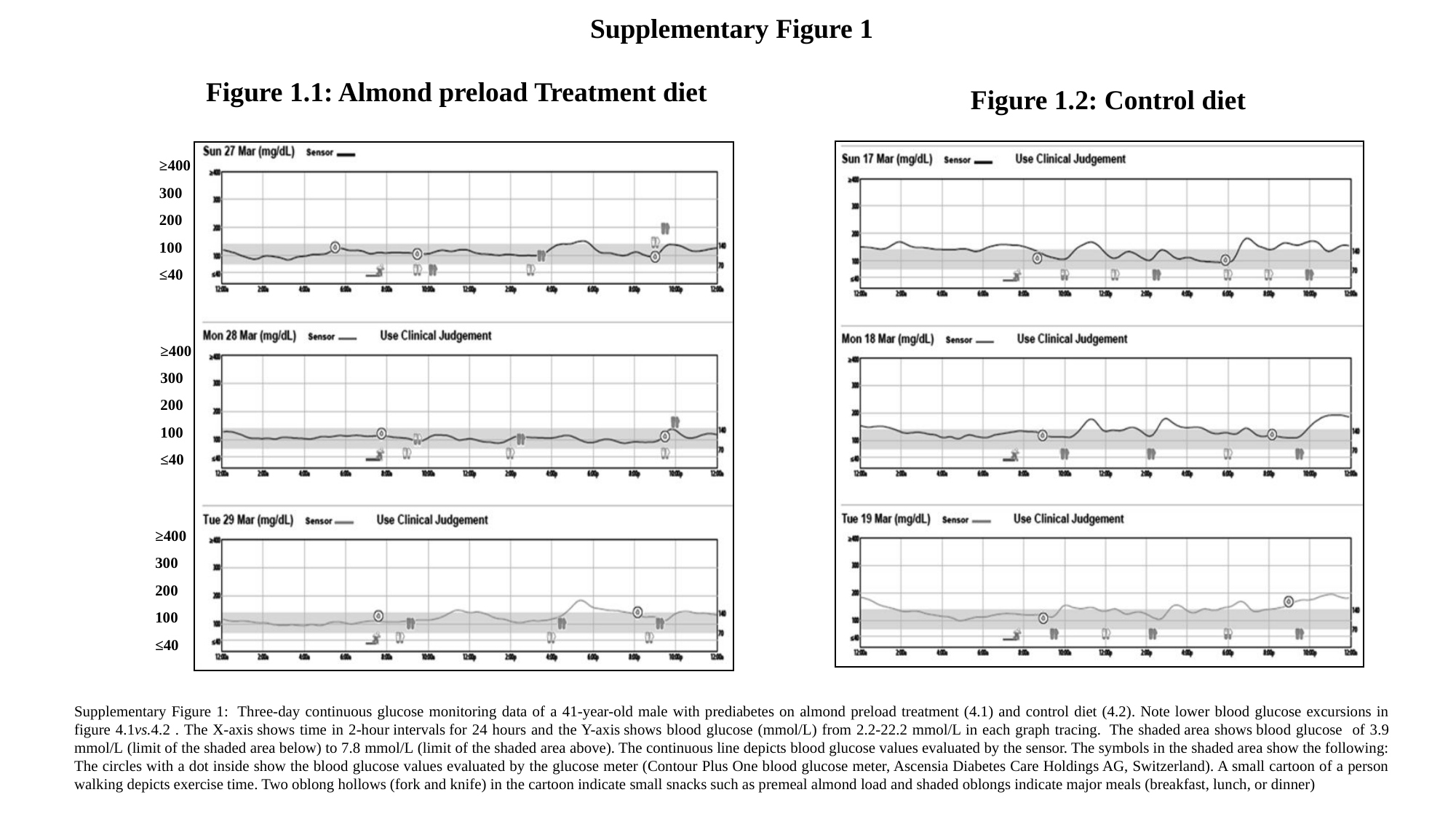

Supplementary Figure 1
Figure 1.1: Almond preload Treatment diet
Figure 1.2: Control diet
≥400
300
200
100
≤40
≥400
300
200
100
≤40
≥400
300
200
100
≤40
Supplementary Figure 1:  Three-day continuous glucose monitoring data of a 41-year-old male with prediabetes on almond preload treatment (4.1) and control diet (4.2). Note lower blood glucose excursions in figure 4.1vs.4.2​ ​. ​The X-axis shows time in ​2-hour ​intervals for 24 hours and ​the ​​Y-axis shows blood glucose (mmol/L) from 2.2-22.2 mmol/L in each graph tracing.  ​The shaded area ​shows blood glucose ​ of ​3.9 mmol/L (limit of the shaded area below) to 7.8 mmol/L (limit of the shaded area above). The continuous line depicts blood glucose values evaluated by the sensor. The symbols in the shaded area show the following: The circles with a dot inside show the blood glucose values evaluated by the glucose meter (Contour Plus One blood glucose meter, Ascensia Diabetes Care Holdings AG, Switzerland). A small cartoon of a person walking depicts exercise time. Two oblong hollows (fork and knife) in ​the ​cartoon indicate small ​snacks such as premeal almond load and shaded oblongs indicate major meals (breakfast, lunch, or dinner)
